# Synthesis of ε-Fe_2–3_N Particles for Magnetic Hyperthermia

**DOI:** 10.3390/jfb16060203

**Published:** 2025-06-01

**Authors:** Soichiro Usuki, Tomoyuki Ogawa, Masaya Shimabukuro, Taishi Yokoi, Masakazu Kawashita

**Affiliations:** 1 Graduate School of Medical and Dental Sciences, Institute of Science Tokyo, 1-5-45 Yushima, Bunkyo-ku, Tokyo 113-8549, Japan; 2 Graduate School of Engineering, Tohoku University, 6-6-5 Aramaki-Aoba, Aoba-ku, Sendai 980-8579, Japan; tomoyuki.ogawa.d1@tohoku.ac.jp; 3 Laboratory for Biomaterials and Bioengineering, Institute of Integrated Research, Institute of Science Tokyo, 2-3-10, Kanda-Surugadai, Chiyoda-ku, Tokyo 101-0062, Japan; shimabukuro.bcr@tmd.ac.jp (M.S.); yokoi.taishi.bcr@tmd.ac.jp (T.Y.)

**Keywords:** iron nitride, ε-Fe_2–3_N, heat-generation property, magnetic hyperthermia

## Abstract

Little research has focused on using iron nitride as thermoseed particles in magnetic hyperthermia, although magnetite (Fe_3_O_4_) is commonly used for this purpose. In the present study, we focus on iron nitride, especially ε-Fe_2–3_N. ε-Fe_2–3_N particles were synthesized from hematite (α-Fe_2_O_3_) and sodium amide (NaNH_2_) under various synthesis conditions, and the heat-generation properties of the particles were investigated to reveal the synthesis conditions that lead to particles with notable heat-generation performance. The particles synthesized at 250 °C for 12 h increased the temperature of an agar phantom by approximately 20 °C under an alternating magnetic field (100 kHz, 125 Oe, 600 s), suggesting that ε-Fe_2–3_N particles can be used for magnetic hyperthermia. The analysis results for the particles synthesized under different conditions suggest that the heat-generation properties of ε-Fe_2–3_N were affected by several factors, including the nitrogen content, particle size, crystallite size, saturation magnetization, and coercive force.

## 1. Introduction

Hyperthermia has been used for various cancer treatments for decades. Several methods are available to heat small diseased areas locally via hyperthermia. Tumors in the liver, kidney, and lungs have been treated with radiofrequency ablation, and deep brain tumors have been treated with laser interstitial thermal therapy [1]. In addition, Fe_3_O_4_-based particles have been developed as thermoseeds for magnetic hyperthermia [1,2], which is a minimally invasive treatment method in which the thermoseeds efficiently heat only the tumor, leading to tumor cell death at a temperature of 43.5–55.5 °C (the average maximum temperature inside the treatment field) in clinical settings when an alternating magnetic field is applied from an external source [3,4,5,6]. However, eradicating tumors using only heating is difficult because no thermoseed is available that exhibits superior heat-generation properties when used in conjunction with available clinical equipment [2,4,7,8]. Currently, combination therapies are recommended because of sensitizing effects of multiple therapies with chemotherapy or radiation therapies [5,6,9,10,11,12]. If thermoseeds with superior heat-generation properties could be developed, they could provide a hyperthermic effect at low doses and maintain this effect with few side effects even if multiple therapies are performed.

Iron nitride has various chemical compositions—α″-Fe_16_N_2_, α′-Fe_8_N, γ′-Fe_4_N, ε-Fe_2–3_N, ζ-Fe_2_N, and γ″-FeN [13,14,15,16,17]. α″-Fe_16_N_2_, γ′-Fe_4_N, and ε-Fe_2-3_N are ferromagnetic with a high saturation magnetization (*M*_s_) [13,15,18,19,20]. The unique characteristics of iron nitride make it suitable for several applications, including corrosion-resistant materials [21,22,23], catalysts [24,25,26], and electrode materials [25,26]. Iron nitride can be synthesized using ammonia gas [13,16,18], molten salt [20,27], or plasma [22,23,28,29] as a source of nitrogen; however, most of the synthesis methods require high energy. A method for synthesizing ε-Fe_2–3_N from iron oxides such as hematite (α-Fe_2_O_3_) or magnetite (Fe_3_O_4_) using sodium amide (NaNH_2_) as a nitrogen source was recently reported [30,31]. The reported magnetic properties of ε-Fe_2–3_N include an *M*_s_ of 59.1 emu/g for Fe_2.8_N at room temperature, and the *M*_s_ for ε-Fe_2–3_N was found to decrease with decreasing Fe/N ratio [32]. Bulk ε-Fe_2_N has an *M*_s_ of approximately 22.2 emu/g [20], while bulk ε-Fe_3_N has a value of 123 emu/g [33], which is higher than that for Fe_3_O_4_ (85.8 emu/g) [34]. However, studies of the heat-generation performance of ε-Fe_2–3_N have been limited [35].

Heat generation by magnetic particles is strongly dependent on the particle size as their magnetic properties vary accordingly. When they are sufficiently small, magnetic particles no longer exhibit hysteresis loss but instead generate heat primarily through Brownian relaxation and Néel relaxation; such particles are referred to as superparamagnetic [2,36]. Fe_3_O_4_ and ε-Fe_3_N particles, for instance, become superparamagnetic at diameters of approximately 16 nm and 15 nm or smaller, respectively [37,38]. As the particle size increases, hysteresis loss becomes the dominant contributor to heat generation. The coercive force (*H*_c_) generally increases with increasing particle size if the particle contains a single magnetic domain but decreases once the particle changes to a multi-domain structure [37,39,40,41]. Therefore, the magnetic properties of particles can be characterized using the magnetization curve, particularly through parameters such as the saturation *M*_s_ and *H*_c_. The hysteresis loss is proportional to the area enclosed by the magnetization curve. A detailed understanding of the relationship between particle size and magnetic properties is essential for optimizing the design of magnetic particles for hyperthermia applications. However, the size-dependent relationship between *M*_s_ and *H_c_* for Fe_2-3_N particles has yet to be elucidated. Clarifying this relationship is particularly challenging as these magnetic parameters are intricately influenced by a range of factors, including crystallite size, particle morphology, and chemical composition [42,43,44,45].

Our previous studies have shown that α″-Fe_16_N_2_ particles are candidate thermoseeds for magnetic hyperthermia because, compared with Fe_3_O_4_ particles, they exhibit better estimated heat-generation properties [46] and similar cytocompatibility [47]. However, whether other iron nitrides have potential applications as thermoseeds for magnetic hyperthermia is unclear. In a study paper, ε-Fe_2–3_N particles were synthesized from α-Fe_2_O_3_ and NaNH_2_, and their heat-generation performance was evaluated to assess their feasibility as thermoseeds for magnetic hyperthermia. Several groups have attempted to prepare iron oxide particles with superior magnetic properties or heat-generation properties by optimizing the preparation conditions; they found that the magnetic properties and heat-generation properties of iron oxide particles are related to their size [48,49,50]. Therefore, we evaluated the heat-generation properties of ε-Fe_2–3_N particles prepared at different temperatures for different durations to investigate the effects of the size, composition, and magnetic properties of the particles on their heat-generation performance. The heat-generation properties of ε-Fe_2–3_N particles were compared with those of commercially available Fe_3_O_4_ particles. We also attempted to identify the experimental conditions for preparing ε-Fe_2–3_N particles whose heat-generation properties make them suitable for magnetic hyperthermia.

## 2. Materials and Methods

### 2.1. Synthesis of Samples

On the basis of the reference study [30], iron nitride particles were synthesized using α-Fe_2_O_3_ (FE010PB, Kojundo Chemical Laboratory Co., Ltd., Saitama, Japan) and 98% NaNH_2_ (208329, Sigma-Aldrich Co. LLC, St. Louis, MO, USA) (1:5 molar ratio). Typically, 1.8 g of mixed powder was loaded into a polytetrafluoroethylene (PTFE) crucible containing carbon fibers (HUTc-25, SAN-AI KAGAKU Co., Ltd., Aichi, Japan). The crucible was placed in a pressure digestion vessel (HU-25, SAN-AI KAGAKU), and the synthesis was conducted in a glovebox filled with N_2_ gas ([O_2_] ≤ 1.0%). The samples were heated at 230–260 °C for 6–96 h in a constant-temperature oven (DKN-302, Yamato Scientific Co., Ltd., Tokyo, Japan). The temperature was raised to the synthesis temperature within a period of 1 h, after which the synthesis was carried out for the desired duration. The pressure digestion vessel was then removed from the oven, cooled at room temperature, and opened in a draft chamber. The synthesized samples were taken from the vessel, washed by stirring in 50 mL of ethanol using a stirrer (1-5473-03, AS ONE Corp., Osaka, Japan) for 10 min, filtered by vacuum filtration, and then dried at room temperature overnight.

### 2.2. Characterization of Samples

The sample morphology, particle size, and particle size distribution were characterized by field-emission scanning electron microscopy (FE-SEM; JSM-7900F, JEOL Ltd., Tokyo, Japan) combined with energy-dispersive X-ray spectroscopy (EDS; JED-2300, JEOL) conducted at an acceleration voltage of 15 kV. The particle size distributions were obtained from FE-SEM images using standard tools implemented in the open-source software Fiji-ImageJ (version 2.14.0/1.54f). The particle size was evaluated by extracting arbitrary particles from the acquired SEM images, and the maximum circumscribed circle was directly measured for each particle. In total, measurements were performed on 300 individual particles. The crystalline phases of the samples were investigated by powder X-ray diffraction (XRD; MiniFlex600, Rigaku Corp., Tokyo, Japan) under the following conditions: Cu-K_α_ (*λ* = 0.15418 nm) line, 40 kV, and 15 mA. The average crystallite size *d* was estimated from the XRD patterns according to the Scherrer equation:

(1) $$d=\frac{K\lambda }{\beta \cos\theta }$$

where *K* is the Scherrer constant (0.9 was chosen in this case), *λ* is the wavelength of the incident beam, *β* is the full-width at half-maximum (FWHM) of the selected peak, and *θ* is the peak position. Data are represented as the mean ± standard deviation (SD) of three independent experiments.

### 2.3. Evaluation of Magnetic Properties of Samples

The magnetic properties of samples were analyzed in the temperature range from −263 to 76.9 °C by vibrating sample magnetometry (VSM; MPMS3 SQUID, Quantum Design Inc., San Diego, CA, USA). The *M*_s_, remanent magnetization (*M*_r_), and coercive force (*H*_c_) for samples were determined from their magnetization hysteresis under a direct current (DC) magnetic field of |*H*| = 30 kOe. Commercially available Fe_3_O_4_ (310069, Sigma-Aldrich, St. Louis, MO, USA) was used as a reference sample.

### 2.4. Measurement of the Heat-Generation Properties of Samples

**Figure 1:**
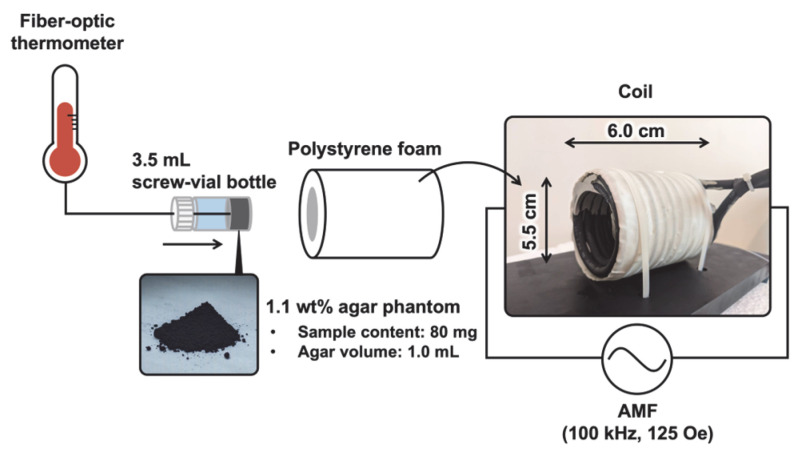
A schematic of the measurement method for heat-generation properties of samples.

The heat-generation properties of each particle sample were evaluated using an alternating magnetic field (AMF) generator (Toyo Electronics Corp., Tokyo, Japan) powered by a DC power supply (ZX-S1600H, TAKASAGO Ltd., Kanagawa, Japan). Each 80 mg particle sample was dispersed in 1.1 wt% agar (FUJIFILM Wako Pure Chemical Corp., Osaka, Japan) with 1.0 mL of ultrapure water, and this liquid was solidified in 3.5 mL screw vial bottles (AS ONE). This bottle was covered in polystyrene foam to prevent it from becoming hot due to the coil (Figure 1). The alternation frequency and magnetic field strength were 100 kHz and 125 Oe, respectively. These AMF conditions were almost equivalent to those used clinically (100 kHz and 25–188 Oe) [5]. Furthermore, the product of the alternation frequency and the magnetic field strength used in this experiment (*f* × *H* = 9.55 × 10^8^ A/(m·s)) remained well within the widely accepted safety limit (*f* × *H* = 5 × 10^9^ A/(m·s)) [7,51]. The temperature of the agar phantom was measured using a fiber-optic thermometer (TMS-G4-10-100ST, Opsens Solutions Inc., Québec, QC, Canada) for 600 s during exposure to the AMF. The temperature increase (Δ*T*) of the agar phantom was estimated by subtracting the temperature of the agar without a sample from that with a sample. Data are represented as the mean ± SD of four independent experiments. Commercially available Fe_3_O_4_ (310069, Sigma-Aldrich) was used as a reference sample.

### 2.5. Statistics

Statistical analyses were performed using Tukey’s honest significance difference (HSD) multi-comparison test implemented with the R language (version 4.3.2) in RStudio (version 2024.04.01+748). The significance level was defined as * *p* < 0.05, ** *p* < 0.01, or *** *p* < 0.001.

## 3. Results

**Figure 2:**
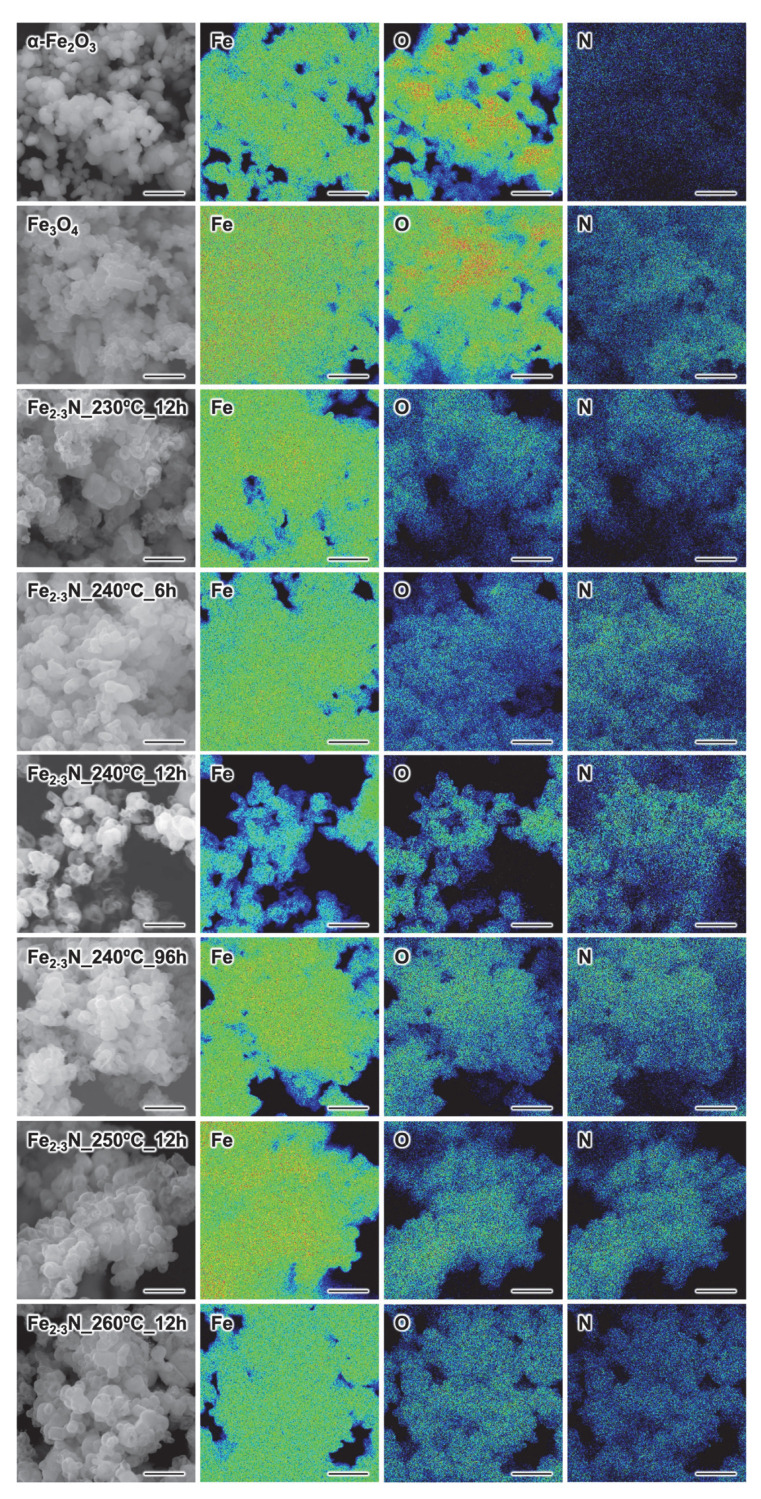
FE-SEM images and corresponding EDS mapping images of samples. Scale bars: 1 µm.

**Figure 3:**
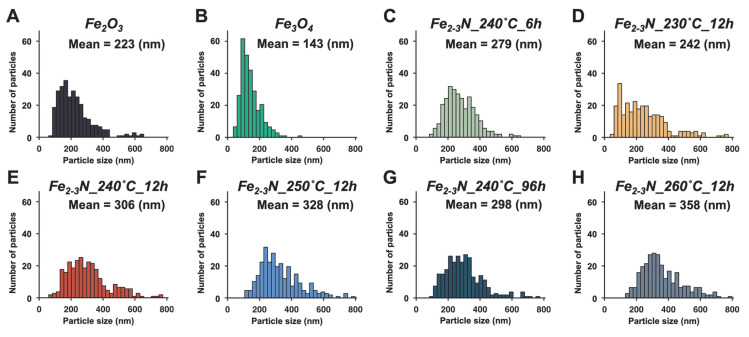
Mean particle sizes for samples (**A**–**H**), as estimated from FE-SEM images. *n* = 300.

FE-SEM images and corresponding EDS mapping images of the α-Fe_2_O_3_ sample, the Fe_3_O_4_ sample, and the synthesized samples are shown in Figure 2. The EDS mapping images represented the distribution of iron, oxygen, and nitrogen on the sample surface. EDS spectra of the representative samples α-Fe_2_O_3_, Fe_3_O_4,_ and Fe_2-3_N_250°C_12h can also be found in Figure S1 in the Supporting Information. The synthesized samples contained nitrogen and iron, and oxygen was also detected (Figure 2 and Figure S1 in the Supporting Information). The samples Fe_2–3_N_230°C_12h, Fe_2–3_N_240°C_6h, Fe_2–3_N_240°C_12h, Fe_2–3_N_240°C_96h, Fe_2–3_N_250°C_12h, and Fe_2–3_N_260°C_12h had rough surfaces, while the surfaces of the α-Fe_2_O_3_ and Fe_3_O_4_ particles were smooth (Figure 2). Additionally, a higher-magnification photograph of Fe_2-3_N_250°C_12h is presented in Figure S2 in the Supporting Information. Figure 3 shows the size distributions for the particles, with the mean particle size also indicated. The samples were composed of spherical particles with diameters of approximately 250–350 nm (Figure 3C–H), and the mean particle size increased with increasing synthesis temperature when the synthesis time was held constant at 12 h (samples Fe_2–3_N_230°C_12h, Fe_2–3_N_240°C_12h, Fe_2–3_N_250°C_12h, and Fe_2–3_N_260°C_12h) (Figure 3D–F,H). However, when the synthesis temperature was held constant at 240 °C (samples Fe_2–3_N_240°C_6h, Fe_2–3_N_240°C_12h, and Fe_2–3_N_240°C_96h), no remarkable difference was observed in the mean particle size between the samples synthesized for 12 h and 96 h (Figure 3C,E,G). The mean particle size for the Fe_3_O_4_ reference sample was 143 nm, and the size distribution was narrower than those for the other samples.

**Figure 4:**
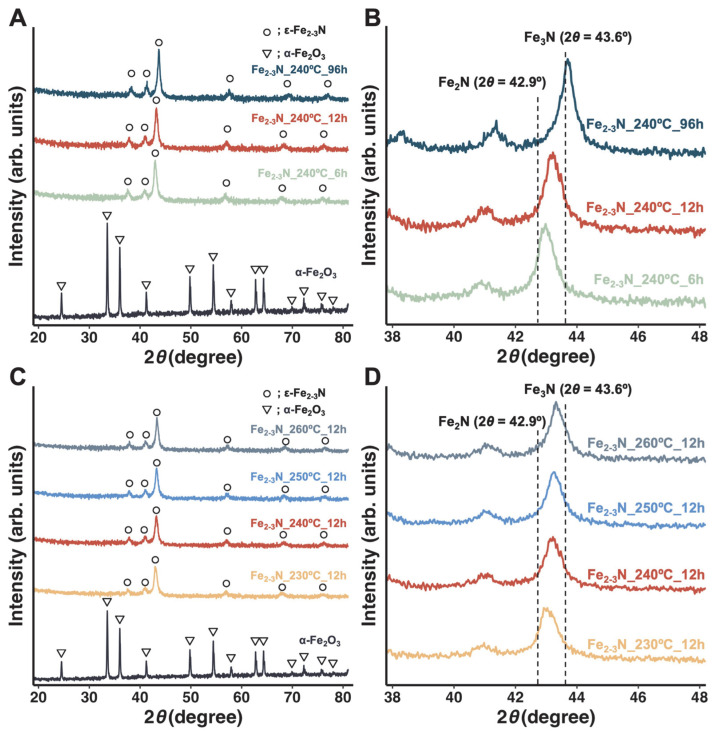
XRD patterns for samples synthesized at 240 °C for different synthesis times (**A**,**B**) and those of samples synthesized at different temperatures for 12 h (**C**,**D**). XRD peak positions for α-Fe_2_O_3_ (PDF No. 00-001-1053), ε-Fe_2_N (PDF No. 01-076-0090), and ε-Fe_3_N (PDF No. 01-083-0877) are shown in Figure 4A,B, while ε-Fe_2_N and ε-Fe_3_N are indicated by dashed lines in Figure 4B,D.

Powder XRD patterns for the samples are shown in Figure 4. As a reference, the XRD pattern for the starting α-Fe_2_O_3_ particles (α-Fe_2_O_3_: PDF No. 00-001-1053) is also shown. The XRD peaks attributable to ε-Fe_2–3_N (ε-Fe_2_N: PDF No. 01-076-0090, ε-Fe_3_N: PDF No. 01-083-0877) were observed in the pattern for all of the samples (Figure 4A,C), and they shifted toward a higher angle as the synthesis time was increased from 6 to 96 h (Figure 4B). A similar peak shift was observed for samples prepared at different temperatures (Figure 4D). The ε-Fe_2–3_N sample, identified by XRD, is known to be ferromagnetic, as reported in previous studies [18,19,20]. The unit-cell parameters for the synthesized samples are presented in Table 1. For the samples prepared at 240 °C (samples Fe_2–3_N_240°C_6h, Fe_2–3_N_240°C_12h, and Fe_2–3_N_240°C_96h), the cell volume decreased from 87.9 to 83.5 Å^3^ when the synthesis time was 96 h. For the samples prepared at a constant synthesis time of 12 h (samples Fe_2–3_N_230°C_12h, Fe_2–3_N_240°C_12h, Fe_2–3_N_250°C_12h, and Fe_2–3_N_260°C_12h), the cell volume decreased from 87.2 to 85.6 Å^3^ when the synthesis temperature was 260 °C.

**Figure 5:**
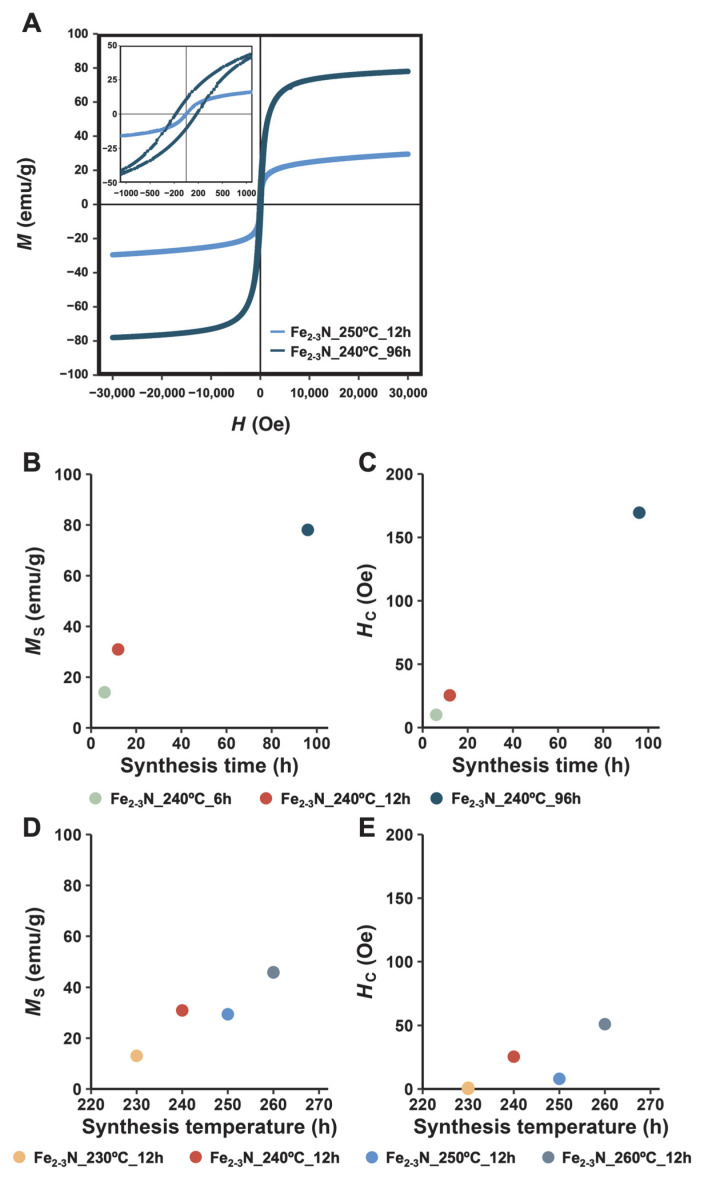
Representative magnetization curves for samples (**A**), magnetic properties of samples synthesized at 240 °C for different synthesis times (**B**,**C**) and samples synthesized at different synthesis temperatures for 12 h (**D**,**E**). The inset shows the expanded low-field region with the magnetic field ranging |*H*| = 1 kOe.

The *M*_s_, *M*_r_, and *H*_c_ values for the samples at 27 °C are shown in Table 2. Figure 5A shows representative magnetization curves for ε-Fe_2–3_N samples synthesized at 240 °C for 96 h and at 250 °C for 12 h where the range of the magnetic field |*H*| is 30 kOe; the inset shows an expanded low-field region in which the range of |*H*| is 1 kOe. The hysteresis curves corresponding to Fe_2–3_N_240°C_96h show a hysteresis loop, whereas those for Fe_2–3_N_250°C_12h do not show hysteresis in the |*H*| range of 1 kOe (Figure 5A). Samples Fe_2–3_N_240°C_96h and Fe_2–3_N_250°C_12h show *M*_s_ values of 78.1 emu/g and 29.4 emu/g, *M*_r_ values of 10.7 emu/g and 0.5 emu/g, and *H*_c_ values of 170 Oe and 8.8 Oe, respectively. Similar trends were observed for the *M*_s_ and *M*_r_ values, with no particular difference noted between the two values. The reference Fe_3_O_4_ particles show an *M*_s_ of 91.6 emu/g, *M*_r_ of 5.5 emu/g, and a *H*_c_ of 71.6 Oe. Figure 5B–E shows the *M*_s_ and *H*_c_ values for samples plotted against the synthesis time and synthesis temperature. The plots indicate that *M*_s_ and *H*_c_ tend to increase with increasing synthesis temperature and increasing synthesis time, except for sample Fe_2–3_N_250°C_12h.

**Figure 6:**
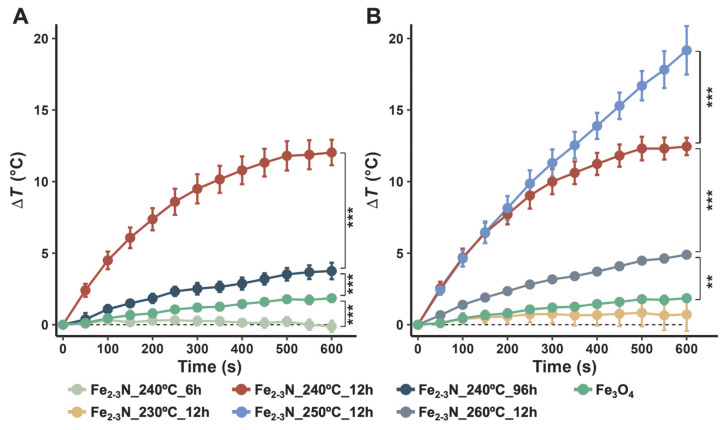
Temperature increase (Δ*T*) in agar phantom with samples dispersed and placed under an alternating magnetic field. The Δ*T* for samples synthesized at 240 °C for different synthesis times and those synthesized at different temperatures for 12 h are shown in (**A**) and (**B**), respectively. Each data point is presented as the mean ± standard deviation (SD) from four independent experiments, and the statistical significance (** *p* < 0.01, *** *p* < 0.001) was computed via Tukey’s HSD test.

The temperature increase (Δ*T*) for the agar phantom in which samples were dispersed and placed under an AMF is shown in Figure 6. Δ*T* for the present samples, except for samples Fe_2–3_N_240°C_6h and Fe_2–3_N_230°C_12h, was higher than that for the Fe_3_O_4_ reference sample. Sample Fe_2–3_N_250°C_12h (19.5°C) exhibited the highest Δ*T* among the investigated samples, and sample Fe_2–3_N_240°C_12h exhibited the highest Δ*T* among the samples synthesized at 240 °C for different synthesis times (Figure 6A).

**Figure 7:**
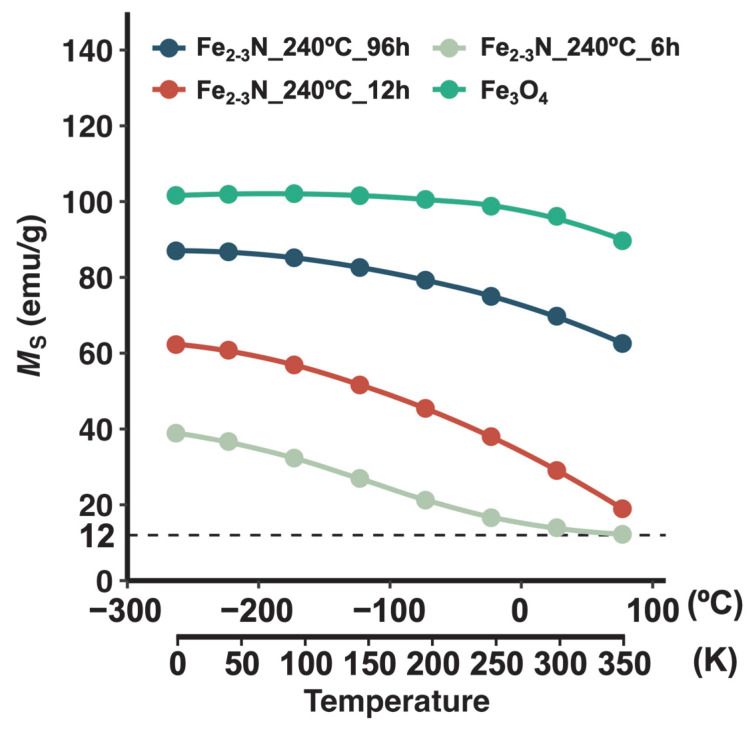
*M*_s_ of samples prepared under different synthesis times, where *M*_s_ was measured at different temperatures.

*M*_s_ curves were acquired for the samples at different temperatures (Figure 7). The *M*_s_ values for the samples and the Fe_3_O_4_ reference sample gradually decreased with increasing temperature. In particular, the *M*_s_ value for sample Fe_2–3_N_240°C_6h converged at ~12 emu/g at ~26.9 °C (~300 K).

**Figure 8:**
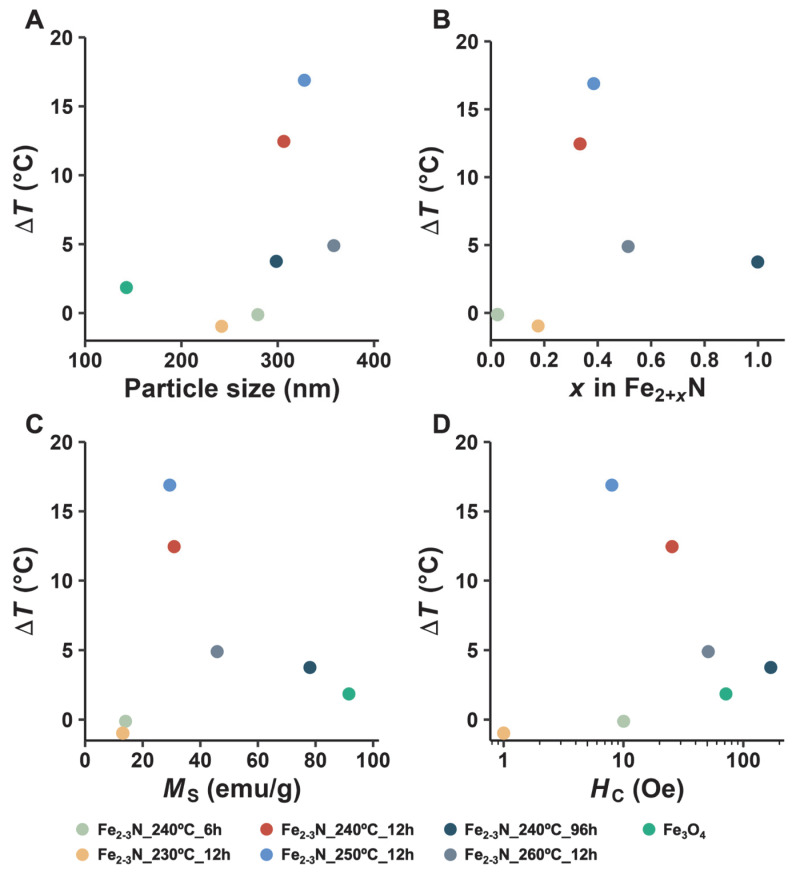
Temperature increase (Δ*T*) in agar phantom plotted against mean particle size (**A**), *x* in ε-Fe_2+*x*_N (**B**), *M*_s_ (**C**), and *H*_c_ (**D**).

Figure 8 shows Δ*T* for the agar phantom in which samples were dispersed versus the mean particle diameter (A), *x* in ε-Fe_2+*x*_N (B), *M*_s_ (C), and *H*_c_ (D). Δ*T* increased with increasing mean particle size up to 328 nm (sample Fe_2–3_N_250°C_12h) and rapidly decreased when the particle size was 358 nm (sample Fe_2–3_N_260°C_12h) (Figure 8A). Δ*T* increased with increasing *x* in ε-Fe_2+*x*_N up to Fe_2_._39_N (sample Fe_2–3_N_250°C_12h) and decreased until Fe_3_._00_N (Fe_2–3_N_240°C_96h) (Figure 8B). Δ*T* was maximal for the sample with an *M*_s_ of 29.4 emu/g; however, no clear relationship was observed between Δ*T* and *H*_c_ for the samples, and the highest Δ*T* was obtained for sample Fe_2–3_N_250°C_12h, which exhibited a *H*_c_ of 8.80 Oe (Figure 8C,D).

## 4. Discussion

In the present study, we synthesized ε-Fe_2–3_N particles at various temperatures and for various synthesis times to elucidate the preparation conditions that yield particles with excellent heat-generation properties. The EDS mapping images indicated that the synthesized samples contained nitrogen as a result of nitridation by NaNH_2_ (Figure 2). Nitrogen was not detected in the α-Fe_2_O_3_ and Fe_3_O_4_ samples (Figure S1 in the Supporting Information); the nitrogen signals in the EDS mapping images are, in fact, artifacts associated with the overlap of the carbon and oxygen peaks in the EDS spectra (Figure S1 in the Supporting Information). All of the samples were composed of crystalline ε-Fe_2–3_N (Figure 4A,C). The XRD peaks for all of the samples were located between those for ε-Fe_2_N and ε-Fe_3_N (Figure 4B,D), and they shifted toward higher angles with increasing synthesis time (Figure 4B). The composition of the synthesized ε-Fe_2+*x*_N was estimated from the unit-cell volume on the basis of the linear calibration model established by O’Sullivan et al. [31] (Table 1 and Figure S3 in the Supporting Information). The unit-cell volume of the samples decreased with increasing synthesis time and increasing temperature. In accordance with the change in unit-cell volume, the estimated composition changed from Fe_2.02_N to Fe_3.00_N for the samples prepared at 240 °C and from Fe_2.18_N to Fe_2.51_N for the samples heated for 12 h, which means that the composition of the samples changed from ε-Fe_2.02_N to ε-Fe_3.00_N by releasing nitrogen during the synthesis. Ma et al. reported that ε-Fe_2_N decomposed into Fe_3_N and Fe_4_N at 450 °C and then decomposed into Fe_4_N at 540 °C under an Ar atmosphere [20]. Also, Zieschang et al. reported that ε-Fe_3_N decomposed to α-Fe when annealed at temperatures greater than 450 °C [18]. These previously reported results support the decomposition of iron nitride in our study and suggest that it decomposed from ε-Fe_2_N to ε-Fe_3_N.

The crystallite sizes for the samples prepared at 230–260 °C for 12 h were estimated from the XRD patterns in Figure 4D using the Scherrer equation (Equation (1)). The crystallite size for iron nitride ranged between 12.1 and 13.5 nm: 12.1 ± 0.36 nm for sample Fe_2–3_N_230°C_12h, 12.9 ± 0.21 nm for sample Fe_2–3_N_240°C_12h, 13.1 ± 0.16 nm for sample Fe_2–3_N_250°C_12h, and 13.5 ± 0.86 nm for sample Fe_2–3_N_260°C_12h. The crystallite size for ε-Fe_2–3_N in the samples increased with increasing synthesis temperature. By contrast, O’Sullivan et al. reported a decrease in the crystallite size of iron nitride with increasing synthesis temperature and synthesis time because of the thermal decomposition of iron nitride [31]. Increasing the synthesis temperature led to an increase in the crystallite size due to sintering among nanosized particles; it also led to the atomistic-scale substitution of oxygen with nitrogen in the crystal structure during the synthesis, which, in turn, led to sample aggregation. Consequently, these factors contributed to the increase in crystallite size. The particle size also increased with increasing synthesis temperature, driven by sintering and recrystallization processes among particles through atomic diffusion (Figure 3D–F,H) to reduce the surface area of the particles and the interfacial area between them. Magnetic aggregation might also promote sample aggregation and, then, sintering and recrystallization at the interface of the particles, resulting in a further increase in both the crystallite and particle sizes.

The *M*_s_ and *H*_c_ of the samples, except for sample Fe_2–3_N_250°C_12h, increased with increasing synthesis time and temperature (Figure 5B–E). In addition, the estimated compositions (Table 1) indicate a decrease in nitrogen content due to a change from ε-Fe_2_._18_N to ε-Fe_2_._51_N with increasing synthesis time. Because the *M*_s_ and *H*_c_ values for ε-Fe_3_N are 134 emu/g and 122 Oe, and those for ε-Fe_2_N are 22.2 emu/g and 55 Oe, respectively [20,33], the increase in *M*_s_ and *H*_c_ for the samples synthesized for longer synthesis times is attributable to the aforementioned structural changes from ε-Fe_2_._02_N to ε-Fe_3_._00_N.

ε-Fe_2–3_N particles with excellent heat-generation properties are candidate thermoseeds for magnetic hyperthermia, and such ε-Fe_2–3_N particles can be synthesized from α-Fe_2_O_3_ and NaNH_2_ under optimized preparation conditions. Notably, the ε-Fe_2–3_N particles prepared at 250 °C for 12 h showed excellent heat-generation properties under an AMF of 100 kHz and 125 Oe (Figure 6). The heating efficiency is commonly evaluated based on the intrinsic loss power (ILP), which is not affected by the measurement conditions. To evaluate the ILP, it is necessary to quantify the specific absorption rate (SAR), which can be calculated using:

(2) $$\mathrm{SAR}\;={\;C}_{f}\frac{m_{f}}{m_{\mathrm{sample}}}\frac{\mathrm{dT}}{\mathrm{dt}}$$

where *m*_f_ is the mass of the tested ferrofluid, *m*_sample_ is the mass of the magnetic particles, and *dT/dt* is the temperature increase rate [52]. The heat capacity of the ferrofluid, *C*_f_, can be approximated as that of water (4.18 J/(g·K)) when the particle content in the colloid is negligible compared to the liquid volume. In this experiment, the slope of the Δ*T* vs. time curve between 0 and 50 s was defined as *dT*/*dt*. The ILP is given by the formula:

(3) $$\mathrm{ILP}\;=\frac{\mathrm{SAR}}{H^{2}f}$$

where *H* is the magnetic field strength, and *f* is the alternation frequency [52]. The estimated values for the sample with the best heat-generation properties (Fe_2–3_N_250°C_12h) and the Fe_3_O_4_ sample are shown in Table 3. The SAR and ILP values for sample Fe_2–3_N_250°C_12h are seen to be significantly higher than those for the Fe_3_O_4_ sample. The ILP value of 0.257 nH m^2^/kg for Fe_2–3_N_250°C_12h is comparable to those reported for commercially available magnetic particles (0.15–4.57 nH m^2^/kg) used for hyperthermia therapy [52,53], and is not particularly high. In fact, Shaw et al. [54] reported a value of 5.30 nH m^2^/kg for superparamagnetic Fe_3_O_4_ particles, whereas Yan et al. [43] found a value of 6.52 nH m^2^/kg for particles with modified shapes. Under the conditions used in the present study, ε-Fe_2-3_N has better heat-generation properties than Fe_3_O_4_ for particles of the same size (Figure 6). Therefore, synthesis of ε-Fe_2-3_N with superparamagnetic properties may result in heat-generation properties that surpass those of Fe_3_O_4_ with comparable particle sizes. The factors affecting the heat-generation performance and behavior of thermoseeds for magnetic hyperthermia may differ substantially from the factors observed under conditions equivalent to those used clinically for magnetic hyperthermia. This situation arises because of the fluctuating predominant influence of Néel and Brownian relaxation on frequency [55,56]. It is therefore challenging to assert that the heat-generation characteristics can be accurately evaluated in a clinical setting. The concentration of samples in the agar phantom (80 mg/mL) was somewhat higher than that in the clinically used magnetic hyperthermia (median dose 31.4 mg/mL) [5]. Reflecting this situation, however, the samples Fe_2–3_N_250°C_12h and Fe_2–3_N_240°C_12h are expected to heat tumor tissue to a temperature greater than 42.5 °C under the assumption that the agar phantom behaves like tumor tissue.

Samples Fe_2–3_N_240°C_6h and Fe_2–3_N_230°C_12h showed a low *M*_s_ and poor heat-generation performance compared with the other samples obtained using the same synthesis temperature or time. The Curie temperature for this sample might be responsible for its low *M*_s_ and poor heat generation because the Curie temperature for ε-Fe_2_N is 250 K (approximately −23 °C) [20], which is remarkably lower than that for ε-Fe_3_N (558 K (~285 °C)) [57]. The *M*_s_ value for the samples gradually decreased with increasing VSM measurement temperature (Figure 7), and the sample Fe_2–3_N_240°C_6h showed a low *M*_s_ of 14.0 emu/g at the VSM measurement temperature of 26.9 °C (~300 K), indicating that this sample exhibits almost no magnetism at room temperature. Therefore, we speculate that samples Fe_2–3_N_240°C_6h and Fe_2–3_N_230°C_12h, which had an ε-Fe_2_N-like structure (Figure 4B,D), exhibited low *M*_s_ values at room temperature (Figure 7) and poor heat-generation performance (Figure 6) because the Curie temperature for these samples may have been near room temperature. As the VSM measurement temperature was increased, the *M*_s_ value for sample Fe_2–3_N_240°C_6h converged to ~12 emu/g, and the same trend was observed for other samples (Figure 7). This suggests that the synthesized samples may have contained multiple phases and not only ε-Fe_2-3_N. Based on the results of ^57^Fe Mössbauer spectroscopy, O’Sullivan et al. reported that ε-Fe_2–3_N synthesized from Fe_3_O_4_ and NaNH_2_ contained ε-Fe_2+*x*_N, γ″-FeN, and oxynitride (FeO_1−*x*_N*_x_*) phases [31]. Furthermore, an X-ray photoelectron spectroscopy study by Miura et al. [30] indicated the presence of O1s peaks in the spectrum of ε-Fe_2–3_N particles. Although no peaks suggesting the formation of oxynitride or other phases were observed in the XRD patterns for the samples (Figure 4A,C), oxynitride might have formed based on the presence of oxygen atoms in the iron nitride, as indicated by the EDS mapping images (Figure 2). Accordingly, magnetism associated with γ″-FeN and the oxynitride was still present at temperatures higher than 23 °C (~250 K), and it converged to ~12 emu/g (Figure 7).

As evident in Figure 8, many complex factors, including particle size (A), nitrogen content (B), *M*_s_ (C), and *H*_c_ (D), affected the heat-generation performance of the samples. Large magnetic particles with multiple magnetic domains generate heat under an AMF by hysteresis loss, whereas small ones with a single magnetic domain generate heat by relaxation loss, suggesting that the heat-generation performance of particles depends on their size [37,39,50]. The literature contains no report of a critical size between single domains and multiple domains in iron nitride. The critical size for iron nitride can be estimated from *M*_s_ using the magnetic flux density (*I*_s_):

(4) $$D=\frac{3\pi }{I_{s}}\sqrt{A{µ}_{0}}$$

where *D* is the critical size, A is the exchange stiffness constant for iron, and μ_0_ is the permeability of a vacuum. The *D* value for samples Fe_2–3_N_240°C_96h and Fe_2–3_N_260d_12h containing ε-Fe_3_N are 86.7 nm and 147 nm, respectively, as determined from Equation (2) under the assumption that the value of *A* is the same as that for iron (*A* = 0.83 × 10^−11^ J/m). Therefore, the ε-Fe_2–3_N particles with sizes of approximately 250–350 nm are likely multi-domain magnetic particles. Thus, we speculated that the ε-Fe_2–3_N particles with hysteresis loss or a high *M*_s_ and *H*_c_ would show better heat-generation properties. Notably, however, the ε-Fe_2–3_N samples with no hysteresis loop, such as sample Fe_2–3_N_250°C_12h (Figure 5A), exhibited the best heat-generation performance among the investigated samples. The heat-generation performance of samples Fe_2–3_N_240°C_96h and Fe_2–3_N_260°C_12h with an ε-Fe_3_N-like structure or with a hysteresis loop (Figure 5A), like those observed at high *M*_s_ and *H*_c_ values (Figure 5B–E), was poor.

The unexpected high heat-generation performance of sample Fe_2–3_N_250°C_12h can be interpreted as follows. First, we estimated the heat generated by samples from the magnetization hysteresis under a DC magnetic field (DMF) (Figure 6); however, the magnetization curves for magnetic particles under an AMF are not always the same as those under a DMF [58,59,60]. Thus, in contrast to our speculation based on the magnetization curve acquired under a DMF, sample Fe_2–3_N_250°C_12h showed excellent heat-generation performance under an AMF. Second, the magnetic domain structure of ε-Fe_2–3_N particles may be responsible for the unexpected high heat-generation performance of sample Fe_2–3_N_250°C_12h. The particle size and crystallite size for sample Fe_2–3_N_250°C_12h were approximately 250–270 nm and approximately 13 nm, respectively, which indicates that the ε-Fe_2–3_N particles have a multi-domain structure. SEM observations revealed that the iron nitride particles had a surface morphology that was very similar to that reported for the flower-like structures observed by Miura et al. [30] and that the ε-Fe_2–3_N particles were brittle and had a wide particle size distribution (Figure 3). Therefore, the results of the present investigation imply that the ε-Fe_2–3_N particles are a mixture of not only single-domain but also multi-domain particles. Conversely, the Fe_3_O_4_ particles (143 nm) in this study might be only multi-domain because the critical size of these particles (i.e., the size at which their magnetic domain changes from a single domain to a multi-domain structure) is *D* = 76 nm [40]. *M*_s_ and *H*_c_ are known to show different behavior between single-domain and multi-domain particles. In the present study, the ε-Fe_2–3_N samples with single-domain and multi-domain magnetic properties could not be simply compared with the Fe_3_O_4_ particles with only multi-domain magnetic properties. Typically, for both single-domain and multi-domain systems, *M*_s_ increases with increasing particle size and eventually becomes constant. In contrast, *H*_c_ decreases as the particle size exceeds the critical size [37,39,40]. As described above, the ε-Fe_2–3_N particles synthesized in the present study, which have multiple magnetic domains, are expected to exhibit an increase or plateau in *M*_s_ and a decrease in *H*_c_ as the particle size increases. Although, as seen in Figure S4, *M*_s_ indeed shows an increase with increasing particle size, no clear decrease is observed for *H*_c_. This is primarily due to compositional changes, particularly variations in nitrogen content, which complicate the observed trend. Thus, in order to elucidate the relationship between particle size and magnetic properties for ε-Fe_2–3_N, it is necessary to establish a method for synthesizing particles with multiple sizes while maintaining the same chemical composition. In addition to particle size, the shape and/or agglomeration of particles may also strongly influence the magnetic and heat-generation properties [37,42,49,61]. This study shows that the unexpectedly high heat-generation performance of ε-Fe_2–3_N might be attributable to the different magnetic domain structure of ε-Fe_2–3_N compared with that of Fe_3_O_4_. To attain ε-Fe_2–3_N with heat-generation properties better than those for sample Fe_2–3_N_250°C_12h, we need to synthesize ε-Fe_3_N with a particle size that results in superparamagnetism. The relationship between particle size, magnetic domain structure, and *H*_c_ of iron nitride is unclear, similar to the case of iron oxide; further investigation is therefore required.

A biological evaluation of the ε-Fe_2–3_N particles is necessary to investigate their feasibility as thermoseeds for magnetic hyperthermia. The literature contains a few in vitro or in vivo evaluations of iron nitrides. For instance, the cytotoxicity of Fe_16_N_2_ against fibroblasts (Rat-1) was reported not to differ from that of Fe_3_O_4_ [47]; in addition, ε-Fe_3_N/Fe_3_O_4_ and ε-Fe_2_N/SiO_2_ core–shell particles were reported to show low cytotoxicity against tumor cells [35,62]. However, the cytotoxicity of ε-Fe_2–3_N particles has not yet been revealed. In addition, the amount of heat generated by ε-Fe_2–3_N particles is related to the particle size, as observed in the present study, and the design of particles is important to retain the particles in tumor tissue. The particle behavior differs from that in tumor tissue or in normal tissue, where there is a dependence on particle size. The smaller particles (⪅5 nm) of iron oxide accumulate in the nucleus, whereas the larger particles (80–100 nm) widely disperse into tumor tissue and remain near the vasculature [63,64,65]. In addition, smaller particles tend to exhibit relatively greater dissolution, inducing toxicity in cells [66]. Even iron oxide, which is considered to exhibit high biocompatibility, has been suggested to exhibit toxicity in a dose-dependent manner [66,67]. Hence, further investigations to find the appropriate size of ε-Fe_2–3_N particles for in vitro and in vivo experiments will need to consider their behavior within cancer tissue and their toxicity to normal tissue.

## 5. Conclusions

Optimized ε-Fe_2–3_N particles with excellent heating properties were synthesized from α-Fe_2_O_3_ and NaNH_2_ under various synthesis times and temperatures. The effects of changes (especially composition and particle size changes) on their heat-generation and magnetic properties were further verified. The particles of the sample Fe_2_._39_N synthesized at 250 °C for 12 h exhibited the best heat-generation performance, and we verified that the heating characteristics of ε-Fe_2–3_N might change to be complexly affected by multiple factors: nitrogen content, particle size, crystal size, *M*_s_, and *H*_c_. Further work, such as a study of the biocompatibility or antitumor effect of ε-Fe_2–3_N, will be needed to exploit the excellent heating potential of these particles.

## Figures and Tables

**Table 1:** Unit-cell parameters and estimated compositions for ε-Fe_2+*x*_N.

Sample	*a* (Å)	*c* (Å)	Volume (Å^3^)	*x* in Fe_2+*x*_N	Estimated Composition
Fe_2–3_N_240°C_6h	4.79	4.41	87.9	0.0241	Fe_2.02_N
Fe_2–3_N_240°C_12h	4.76	4.40	86.5	0.334	Fe_2.33_N
Fe_2–3_N_240°C_96h	4.70	4.36	83.5	0.999	Fe_3.00_N
Fe_2–3_N_230°C_12h	4.78	4.41	87.2	0.177	Fe_2.18_N
Fe_2–3_N_250°C_12h	4.76	4.40	86.2	0.385	Fe_2_._39_N
Fe_2–3_N_260°C_12h	4.75	4.39	85.6	0.514	Fe_2.51_N

**Table 2:** *M*_s_, *M*_r_, and *H*_c_ values for samples.

Sample	*M*_s_ (emu/g)	*M*_r_ (emu/g)	*H*_c_ (Oe)
Fe_3_O_4_	91.6	5.5	71.6
Fe_2–3_N_240°C_6h	14.0	0.1	10.0
Fe_2–3_N_240°C_12h	30.9	1.2	25.4
Fe_2–3_N_240°C_96h	78.1	10.7	170
Fe_2–3_N_230°C_12h	13.0	−	−
Fe_2–3_N_250°C_12h	29.4	0.5	8.8
Fe_2–3_N_260°C_12h	45.9	3.6	51.0

−: under detection limit.

**Table 3:** Calculated values of SAR and ILP for samples under 100 kHz and 125 Oe.

Sample	SAR (W/g)	ILP (nH m^2^/kg)
Fe_2–3_N_250°C_12h	2.54	0.257
Fe_3_O_4_	0.126	0.0127

## Data Availability

The raw data supporting the conclusions of this article will be made available by the authors on request.

## Supplementary Materials

The following supporting information can be downloaded at https://www.mdpi.com/article/10.3390/jfb16060203/s1: Figure S1: EDS spectra of samples α-Fe_2_O_3_, Fe_3_O_4,_ and Fe_2-3_N_250°C_12h; Figure S2. FE-SEM images of sample Fe_2-3_N_250°C_12h; Figure S3: Dependence of unit-cell volume on stoichiometry *x* of ε-Fe_2+*x*_N assuming a linear dependence; Figure S4: Mean sample particle size plotted against *M*_s_ (A) and *H*_c_ (B).
