# Supplementary material for: Synthesis of ε-Fe2–3N Particles for Magnetic Hyperthermia
Source: J Funct Biomater. 2025 Jun 1;16(6):203. doi: 10.3390/jfb16060203 (PMC12193898; doi:10.3390/jfb16060203)
Supplement: Supplementary file 1 [file jfb-16-00203-s001.zip › jfb-3603504-supplementary.pdf]

Journal of
Functional Biomaterials

Article

Synthesis of ε-$Fe_{2–3}N$ Particles for Magnetic Hyperthermia

Soichiro Usuki [1,*], Tomoyuki Ogawa [2], Masaya Shimabukuro [3], Taishi Yokoi [3] and Masakazu Kawashita [3,*]

1 Graduate School of Medical and Dental Sciences, Institute of Science Tokyo, 1-5-45 Yushima, Bunkyo-ku, Tokyo 113-8549, Japan
2 Graduate School of Engineering, Tohoku University, 6-6-5 Aramaki-Aoba, Aoba-ku, Sendai 980-8579, Japan; tomoyuki.ogawa.d1@tohoku.ac.jp
3 Laboratory for Biomaterials and Bioengineering, Institute of Integrated Research, Institute of Science Tokyo, 2-3-10, Kanda-Surugadai, Chiyoda-ku, Tokyo 101-0062, Japan; shimabukuro.bcr@tmd.ac.jp (M.S.); yokoi.taishi.bcr@tmd.ac.jp (T.Y.)
* Correspondence: usuki.soichiro@tmd.ac.jp (S.U.); kawashita.bcr@tmd.ac.jp (M.K.)

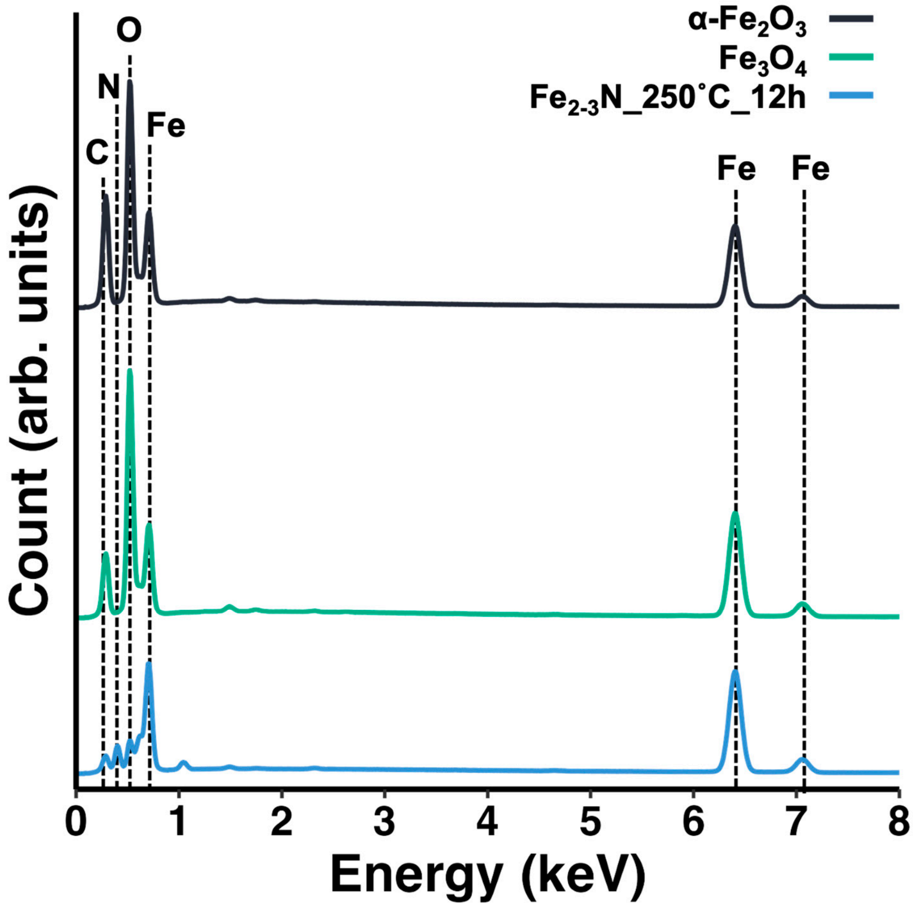

Figure S1. EDS spectra of samples α-Fe_2O_3, Fe_3O_4 and $Fe_{2\text{-}3}N$_250˚C_12h.

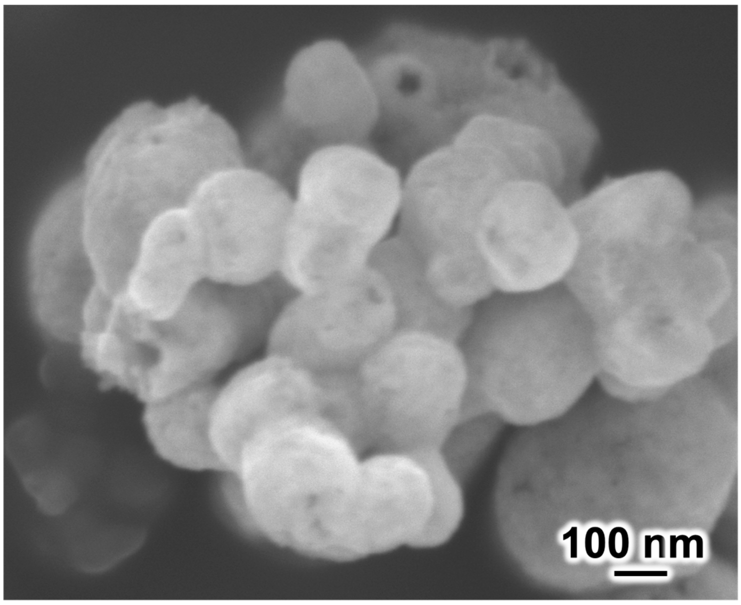

Figure S2. FE-SEM image of sample $Fe_{2\text{-}3}N$_250˚C_12h.

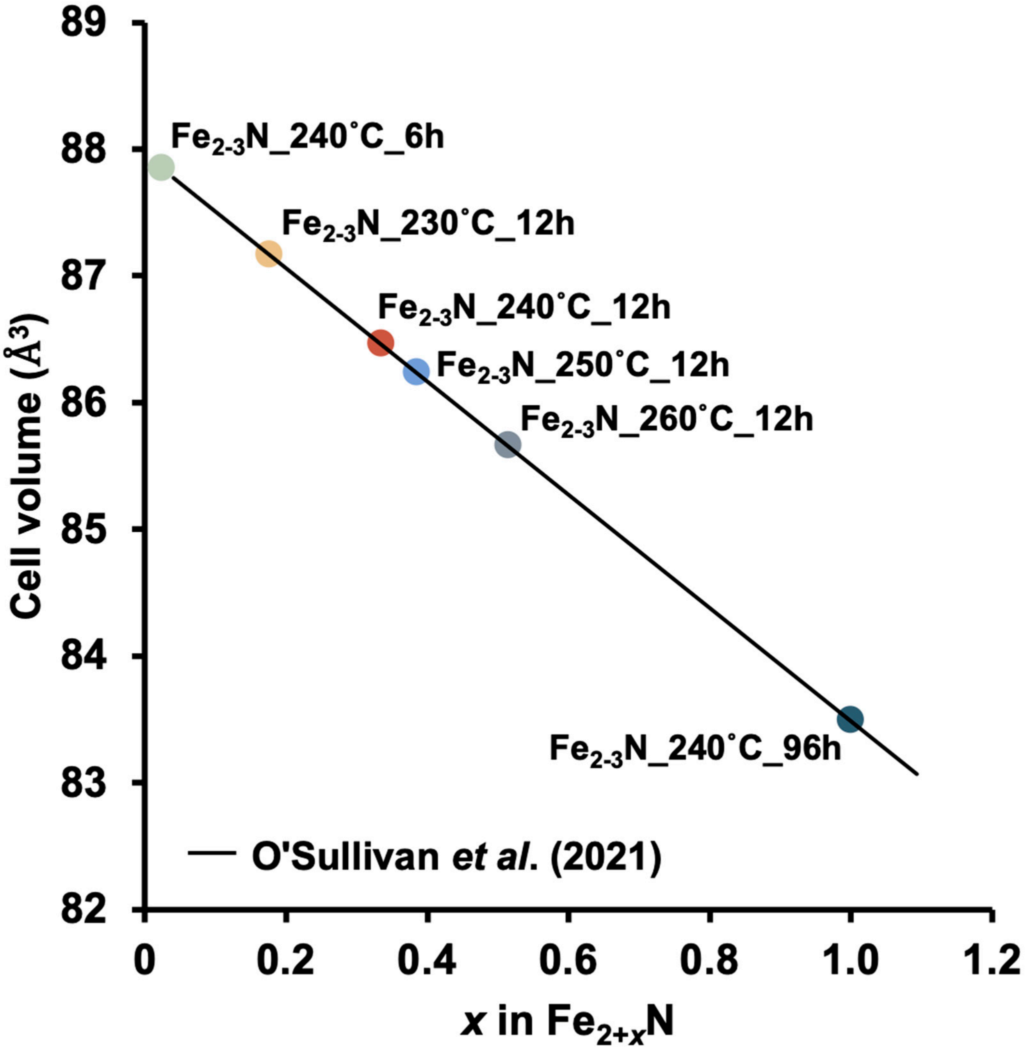

Figure S3. Dependence of the unit-cell volume on stoichiometry x of ε-$Fe_{2+x}N$ assuming a linear dependence. The linear calibration equation $y = -4.47x + 87.96$ was established by O'Sullivan *et al*. [31].

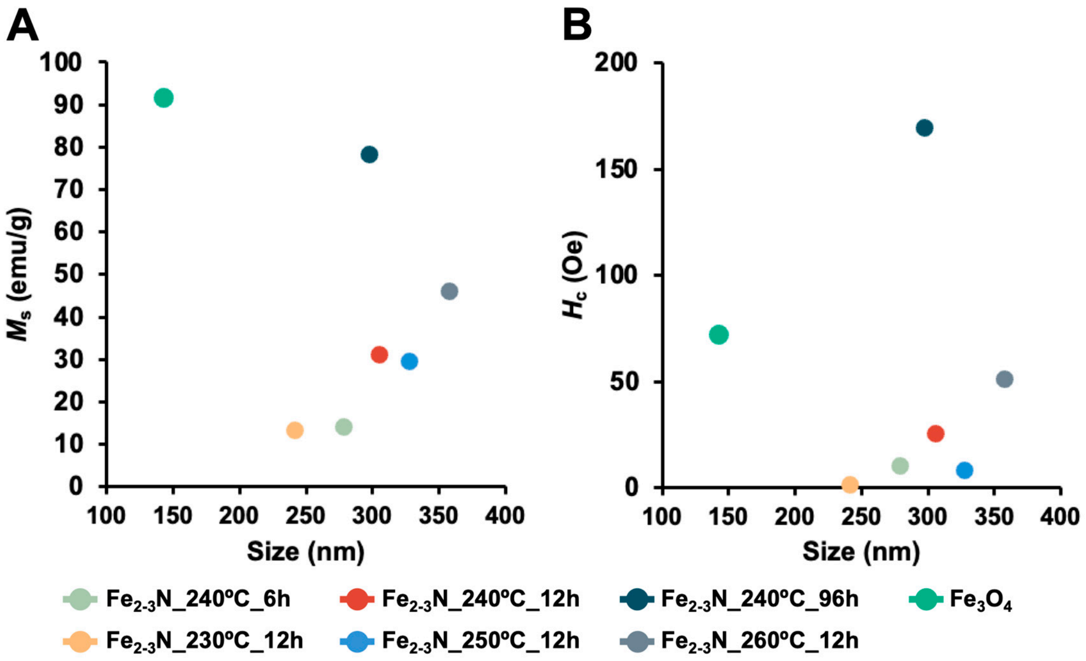

Figure S4. Mean particle size of sample against M_s (A) and H_c (B).
